# The multiple systemic artery to pulmonary artery fistulas resulting in severe irreversible pulmonary arterial hypertension in patient with previous history of pneumothorax

**DOI:** 10.1186/s12890-019-0832-8

**Published:** 2019-04-16

**Authors:** Wojciech Jacheć, Andrzej Tomasik, Marcin Kurzyna, Radosław Pietura, Adam Torbicki, Jan Głowacki, Ewa Nowalany-Kozielska, Celina Wojciechowska

**Affiliations:** 1 2nd Department of Cardiology, School of Medicine with Dentistry Division in Zabrze, Medical University of Silesia, 10 Curie-Sklodowska str, 41-808 Zabrze, Poland; 2 Department of Pulmonary Circulation, Thromboembolic Diseases and Cardiology, Centre of Postgraduate Medical Education, European Health Centre Otwock, 14/18 Borowa str, 05-400 Otwock, Poland; 3 Department of Radiography Medical, University of Lublin, Staszica 11 str., 20-081, 20-954 Lublin, Poland; 4 Department of Radiology and Nuclear Medicine, School of Medicine with Dentistry Division in Zabrze, Medical University of Silesia, 13-15 3-go Maja str, 41-800 Zabrze, Poland

**Keywords:** Systemic artery to pulmonary artery fistulas, Pulmonary arterial hypertension, Pneumothorax

## Abstract

**Background:**

Systemic artery to pulmonary artery fistulas (SA-PAFs), are extremely rare in people without congenital heart disease. In this group of patients pulmonary arterial hypertension was reported in the single case. Then, we describe a case of multiple SA-PAFs, which were the cause of severe nonreversible arterial pulmonary hypertension in a patient who had a right-sided pneumothorax 35 years earlier.

**Case presentation:**

52-year-old male Caucasian patient with echocardiographically confirmed pulmonary hypertension (PH) was admitted to cardiology department due to exertional dyspnea and signs of right ventricle failure. Routine screening for causes of secondary PH was negative. Right heart catheterization (RHC) confirmed a high degree arterial PH [mean pulmonary artery pressure (mPAP); 50,6 mmHg, pulmonary wedge pressure (PWP); 11,3 mmHg, pulmonary vascular resistance (PVR); 11,9 Wood’s units (WU)] irreversible in the test with inhaled nitric oxide. Oxygen saturation (SaO_2_) of blood samples obtained during the first RHC ranged from 69.3 to 73.2%. Idiopathic pulmonary arterial hypertension was diagnosed. Treatment with inhaled iloprost and sildenafil was initiated.

Control RHC, performed 5 months later showed values of mPAP (59,7 mmHg) and PVR (13,4 WU) higher in comparison to the initial measurement, SaO_2_ of blood obtained during RHC from upper lobe artery of the right lung was elevated and amounted 89.7%.

Then, pulmonary arteriography was performed. Lack of contrast in the right upper lobe artery with the evidence of retrograde blood flow visible as a negative contrast in the right pulmonary artery was found. Afterwards, right subclavian artery arteriography detected a huge vascular malformation communicating with right upper lobe artery. Following computed tomography angiogram (angio-CT) additionally revealed the enlargement of bronchial arteries originated fistulas to pulmonary artery of right upper lobe.

In spite of intensive pharmacological treatment, including the therapy of pulmonary hypertension and percutaneous embolisation of the fistulas, the patient’s condition continued to deteriorate further. He died three months after embolisation due to severe heart failure complicated by pneumonia.

**Conclusion:**

Non-congenital SA-PAFs are extremely rare, however, they should be excluded in patients with pulmonary arterial hypertension and history of inflammatory or infectious disease of the lung and pleura, pneumothorax, cancer or Takayashu’s disease and after chest trauma.

## Background

Systemic artery to pulmonary artery fistulas (SA-PAFs) are known to develop in patients with congenital heart diseases, which are combined with right ventricular outflow or pulmonary artery obstruction and in many cases it may be the only blood supply to pulmonary circulation [1].

SA-PAFs are extremely rare in people with normal pulmonary circulation. They can be congenital and non-congenital. Non-congenital fistulas can develop in patients with inflammatory or infectious disease of the lung and pleura, cancer, Takayasu’s disease, or after trauma [2–4]. Congenital SA-PAFs can be detected in patients without any of the above-mentioned risk factors [5].

SA-PAF can be single or multiple and connect pulmonary vascular bed with left internal mammary artery, left subclavian artery, pericardiacophrenic branch of left inferior phrenic artery, left bronchial artery, gastric arteries or others [5, 6]. SA-PAFs are usually asymptomatic. Some patients complained about dyspnea, hemoptysis or symptoms of congestive heart failure [7–11]. The natural outcome of SA-PAFs is not well-known yet. SA-PAFs may be managed by embolisation, resection, or observation [1].

Only in two reported cases of congenital SA-PAFs mild elevated systolic pulmonary artery pressure (to 37 mmHg) was detected [5] and severe pulmonary arterial hypertension was reported in one case of congenital SA-PAFs [12].

We describe a case of non-congenital multiple SA-PAFs which were the cause of severe precapillary pulmonary hypertension in a patient who had right-sided pneumothorax thirty years earlier.

## Case presentation

A 52-year-old male patient with initial diagnosis of pulmonary arterial was admitted to the Department of Cardiology for medical assessment and decision on further treatment. The patient had a history of right-sided spontaneous pneumothorax, treated with thoracentesis and vacuum drainage 35 years earlier. The patient felt increasing dyspnea on exertion. He also complained about signs of right ventricle failure and cyanosis. Four years earlier the patient was hospitalized three times for paroxysmal atrial fibrillation that was converted to sinus rhythm by electric cardioversion besides electrocardiographic examination was normal.

In the same year coronary angiography indicated normal coronary arteries. Echocardiography revealed mild pulmonary hypertension with calculated right ventricle systolic pressure of 36 mmHg without right ventricle dilation.

In the following years, progressive heart failure with increasing pulmonary artery pressure in echocardiography has been observed. Angio-CT was performed on two occasions and chronic thromboembolic pulmonary hypertension was excluded each time. High-resolution computed tomography has excluded interstitial pulmonary pathology. Spirometry was normal. Screening for autoimmune diseases and HIV infection was negative (Table 1).

**Table 1:** Timeline – history, course of the disease, diagnosis and treatment

1976	right-hand pneumothorax
2007	Hospitalized three times for atrial fibrillation and heart failure echocardiography, cardioversion, coronarography were performed. Every time discharged from the departments with diagnosis: Atrial fibrillation, Heart failure, NYHA class II.
2010–2011	Increasing growing shortness of breath and symptoms of heart failure. Patient was hospitalized 3 times. Indirect features of pulmonary hypertension in echocardiography were detected. Three times chest-CT examination was performed; pulmonary embolism or chronic thromboembolic pulmonary arterial hypertension (2 x angio-CT) or pulmonary fibrosis (high resolution computer tomography) was excluded. Diagnosis of pulmonary hypertension was established, WHO class III.
DEC-2011	Admission to cardiology clinic – echocardiography (TTE, TEE), 6-min walking test, NT-proBNP, right heart catheterisation, pulmonary vasoreactivity test. Diagnosis of irreversible arterial pulmonary hypertension was established. Treatment with the illoprost and sildenafil has started
JAN-2012	control visit – improvement in WHO class, decrease of NT-proBNP concentration and increase of 6-min test distance
MAY-2012	Control RHC. SaO_2_ of blood samples obtained during RHC from upper lobe artery of the right lung amounted 87%. Angiographic diagnostic of pulmonary arteries revealed PAH fistulas between subclavian and upper lobe of right lung arteries
JUN 2012	angio-CT of systemic arteries revealed additional presence of bronchial artery fistulas to upper lobe of right lung arteries
III-2013	Embolisation of fistulas
VI-2013	Death as a result of worsening of heart failure combined with pneumonia.

At admission to our clinic functional class of patient was assessed as WHO III, In physical examination the second heart sound (S2) was accentuated with widened split S2. Moreover, heart auscultation indicated holosystolic murmur of tricuspid regurgitation. The auscultation of the chest did not show a vascular murmur. Peripheral and central cyanosis was marked. Hepatomegaly, peripheral edema and varicose veins bilaterally were examined.

SaO_2_ obtained by pulse oximetry method was reduced to 88%. Electrocardiography revealed atrial fibrillation, right axis deviation and negative T wave in precordial leads v1-v3. Additional tests were as follows: NT-proBNP concentration – 3383 pg/ml, six-minute walking test distance - 373 m with 7/10 points in Borg dyspnea score.

**Fig. 1:**
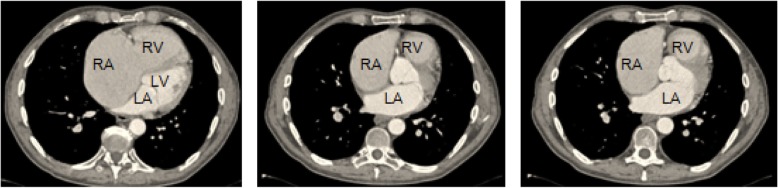
“Angio-CT - preserved continuity of the atrial septum, high degree enlargement of right atrium and ventricle”. *RA* right atrium, *RV* right ventricle, *LA* left atrium, *LV* left ventricle

Echocardiography showed features of severe pulmonary hypertension: right ventricle enlargement to 44 mm (four chamber view), with depressed function of right ventricle TAPSE 9 mm, right atrium area enlargement to 40 cm^2^, severe tricuspid valve regurgitation (++++), increased calculated RVSP to 112 mmHg, shortened pulmonary artery acceleration time to 60 ms and mild pericardial effusion. There was no interventricular septum defect. Trans-esophageal echocardiography did not show a defect in the atrial septum too, which was confirmed later by angio-CT (Fig. 1).

SaO_2_ of blood obtained from radial artery was reduced to 87,8%. Right heart catheterization was performed. SaO_2_ of blood samples obtained during RHC from vena cava superior, vena cava inferior, right atrium, pulmonary trunk, middle lobe artery of the right lung and left pulmonary artery amounted respectively: 64,8%; 77,4%; 73,2%; 70,2%, 69,3%; 71,2% and did not indicate the presence of a left-to-right shunt.

Hemodynamic measurements showed severe precapillary pulmonary hypertension, irreversible in vasoreactivity testing with inhaled nitric oxide (80 ppm) and a combination of oral sildenafil (50 mg) and inhaled nitric oxide (80 ppm) [13] (Table 2).

**Table 2:** Results of pulmonary arterial hypertension reversibility test

	PAPs/PAPd (mPAP) [mm Hg]	ABPs/ABPd (mABP) [mm Hg]	PWP [mm Hg]	PVR [WU]	SVR [WU]	RA [mm Hg]	CI [l/min./m^2^]	SaO_2_ [%]
Baseline	69,8/41,0 (50,6)	104,0/80,0 (88,3)	12,3	11,9	24,4	10,0	1,5	87,8
NO/O_2_ (80 ppm)	63,0/35,6 (44,7)	101,0/74,0 (83)	12,0	9,1	20,8	8,0	1,7	99,0
Sildenafil (50 mg)	68,0/37,6 (47,7)	99,5/70,0 (79,8)	12,0	8,1	16,3	8,0	2,0	84,0
NO/O_2_ (80 ppm)	62,0/34,4 (43,6)	98,0/72,0 (80,3)	12,5	6,9	16,2	8,0	2,1	95,2
Control RHC	75,4/51,8 (59,7)	104,0/78,0 (86,7)	13,5	13,4	21,7	12,0	1,6	90,1

*PAPs* systolic pulmonary artery pressure, *PAPd* diastolic pulmonary artery pressure, *mPAP* mean pulmonary artery pressure, *ABPs* systolic arterial blood pressure, *ABPd* diastolic arterial blood pressure, *mABP* mean arterial blood pressure, *PWP* pulmonary wedge pressure, *PVR* pulmonary vascular resistance, *SVR* systemic vascular resistance, *RAP* right atrium pressure, *CI* cardiac index, *SaO*_*2*_ systemic blood oxygenation, *NO* nitric oxide, *O*_*2*_ oxygen, *ppm* parts per million, *RHC* right heart catheterization

Based on the whole clinical data and the results of RHC, a diagnosis of idiopathic irreversible pulmonary hypertension was established.

Treatment with inhaled iloprost (Ventavis) 6 × 5 μg and sildenafil (Revatio) orally 3 × 20 mg was initiated. Moreover, digoxin (0,1 mg daily), warfarin (INR range: 2–3), furosemide (40 mg orally daily) and spironolactone (100 mg daily) were administered.

Noninvasive follow up was performed one month later – the patient reported improved physical capacity, class II WHO, NT-proBNP concentration: 1014 pg/ml. Six-minute walking test distance - 471 m.

Invasive assessment was done five months later. RHC showed higher than in initial measurements values of PAP [75,4/51,8 (59,7) mm Hg] and PVR (13,4 WU) (Table 2 – control RHC).

Oxygen saturation of blood samples obtained during RHC from upper lobe artery of the right lung was elevated and amounted 89.7%.

**Fig. 2:**
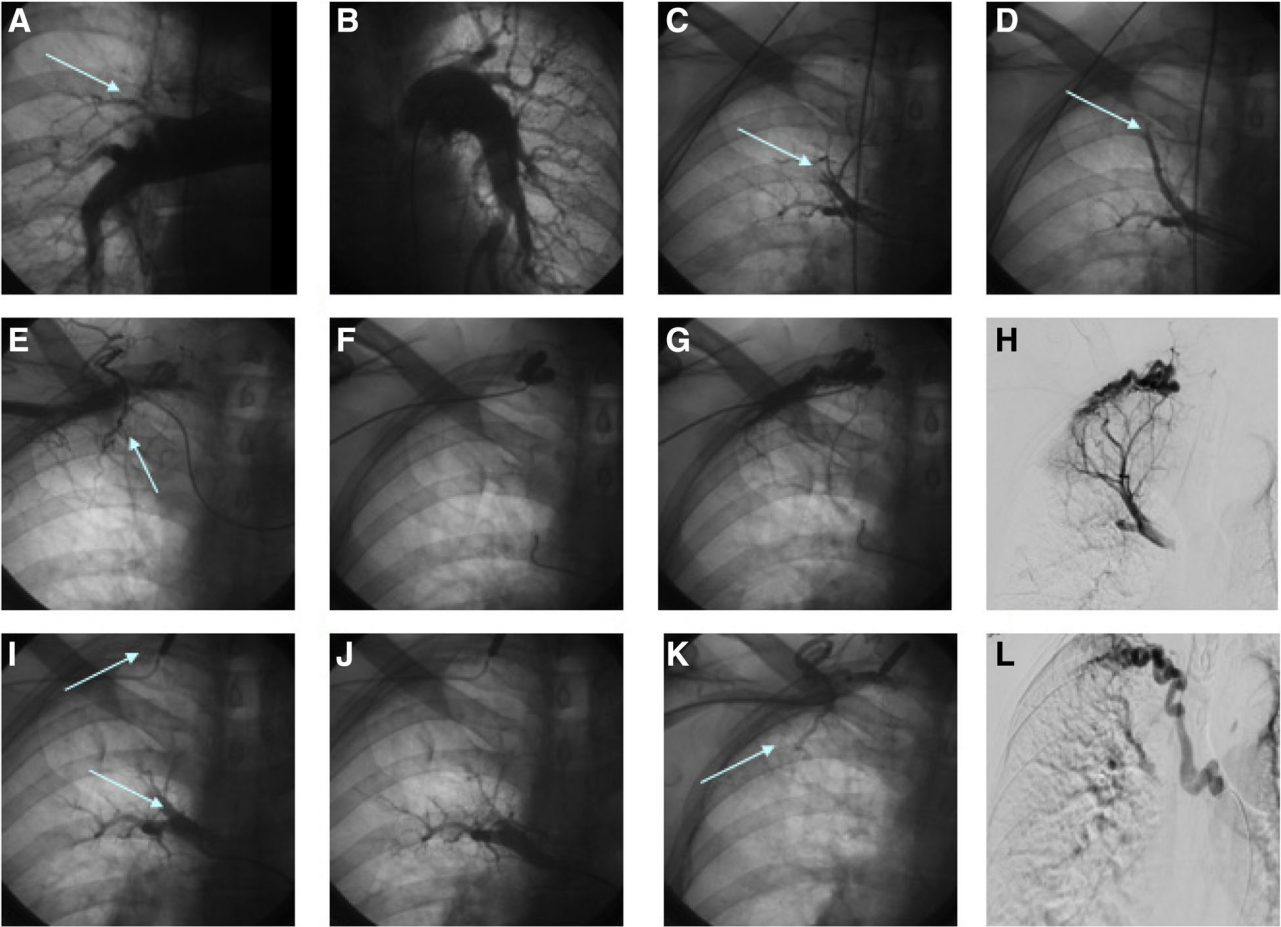
“Pulmonary angiography results. Retrograde filling of the right pulmonary artery is seen, representing fistulas between the subclavian and bronchial arteries and pulmonary artery”. **a** – right pulmonary artery; no contrast of the upper lobe arteries (arrow), **b** – left pulmonary artery, **c**, **d** – selective angiography of the upper lobe artery, visible contrast leaching (arrow) and lack of venous phase, **e** – selective angiography of the right subclavian artery, visible vascular malformation (arrow), **f**, **g**, **h** – selective angiography of fistula between left subclavian artery and pulmonary upper lobe artery, **i**, **j** – occlusion of fistula by balloon (5.0 20 mm), with subsequent selective angiography of the upper lobe artery, still visible contrast leaching (arrow) and lack of venous phase, **k** – selective angiography of the right subclavian artery, visible second vascular malformation (arrow), **l** – pulmonary angiography with contrast injected into an enlarged bronchial artery. Retrograde filling of the right pulmonary artery is seen, representing a fistula between the bronchial artery and pulmonary artery

In the pulmonary angiography reduction of peripheral vascular drawing typical of pulmonary arterial hypertension and the lack of contrast of the right upper lobe artery were detected. Additionally the evidence of retrograde blood flow visible as a negative contrast in right pulmonary artery was found (Fig. 2 a-d). Afterwards, right subclavian artery arteriography was performed and a huge vascular malformation communicating with right upper lobe artery (Fig. 2e-k) was detected.

**Fig. 3:**
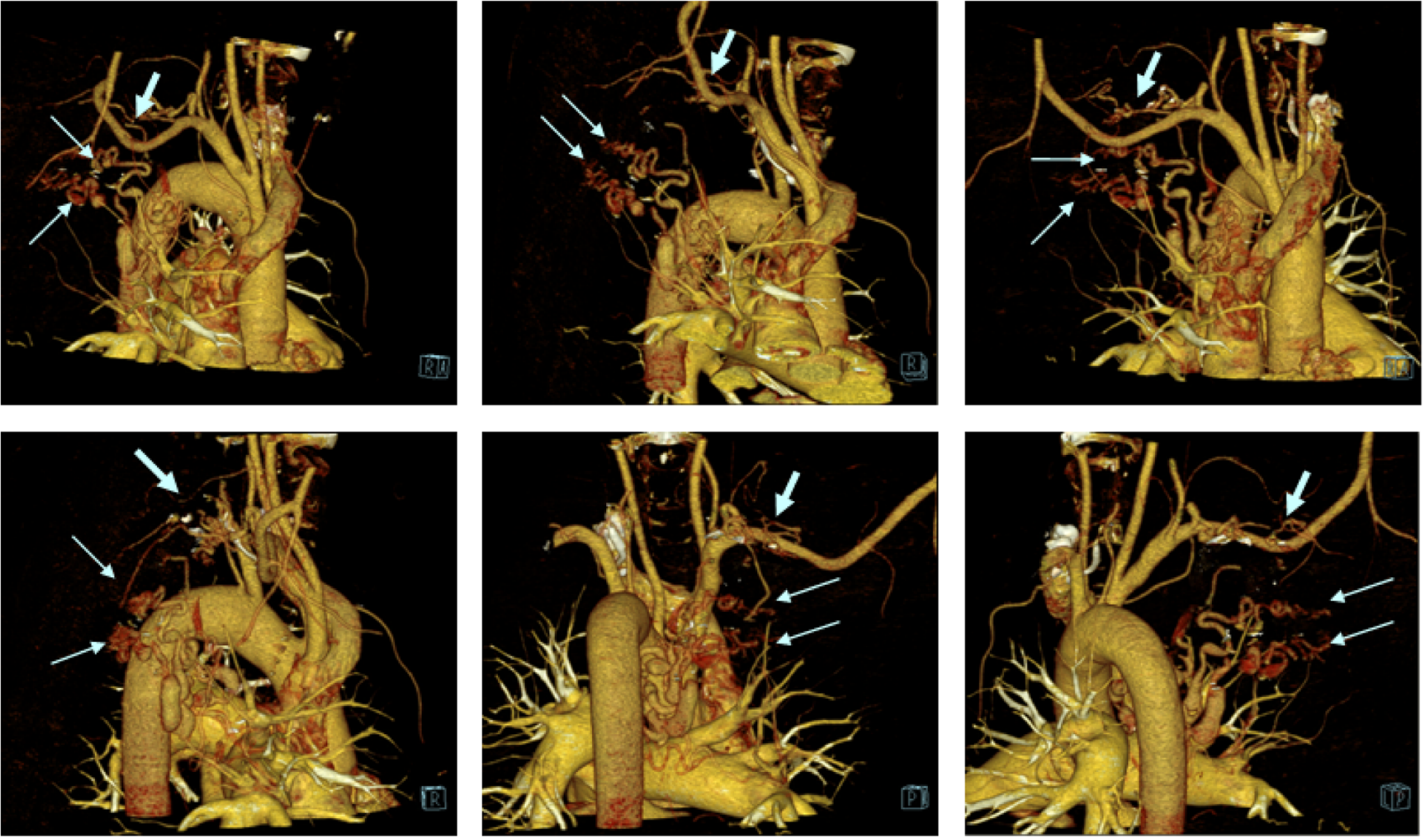
Systemic artery to pulmonary artery fistulas, CT angiography reconstruction; bronchial arteries fistulas (narrower arrows) and subclavian artery fistulas (thicker arrow)

Angio-CT of systemic arteries confirmed presence of previously described fistulas and revealed additional presence of bronchial artery fistulas to upper lobe of right lung arteries (Fig. 3).

Selective embolisation of fistulas was performed (50% mixture of Lipiodol and monomeric n-butyl-2-cyanoacrylate glue). Embolisation did not bring any clinical change in the patient’s condition.

## Discussion and conclusions

A case of 52-year-old male patient with high-grade pulmonary arterial hypertension leading to severe right ventricle failure that was irreversible in vasoreactivity testing with inhaled nitric oxide and sildenafil was reported. Patient had a right-sided pneumothorax 35 years earlier.

Multiple artery-to-artery fistulas between systemic and pulmonary circulation, existing despite normal morphologic development of pulmonary circulation were the cause leading to severe pulmonary hypertension.

During the last four years of his life, the patient presenting with cyanosis and echocardiographic evidence of pulmonary hypertension and had normal spirometry results, HRCT and angio-CT of pulmonary arteries.

Mild to moderate hypoxemia is common in arterial pulmonary hypertension patients [14, 15].

It may be possibly related to ventilation/perfusion mismatch [16], low diffusion capacity, low mixed venous PvO_2_ [16, 17], and sometimes existing right-to-left shunting, which is classically considered to arise from the reopening of patent foramen ovale [14, 18]. In this case normal chest radiographs, HRCT of the chest, and lack of pulmonary edema during vasodilatatory therapy excluded pulmonary veno-occlusive disease as the cause of hypoxemia [19].

We believe that in this case the probable cause of pulmonary hypertension are multiple systemic to pulmonary artery fistulas which initially **cause left-**to**-right** shunt, induce an increase in pulmonary vascular resistance which leads to an increase in pulmonary vascular resistance which eventually results in reversal of the direction of the shunt. It was responsible for the proper perfusion of the upper lobe of right lung during first two angio-CT.

In contrast, in the first RHC the ratio of mean pulmonary to systemic pressure was about 5/8, which clearly indicates the left-to-right direction of the shunt. This may be related to worsening function of right ventricle.

At the stage of the first RHC, we had no premises to extend the diagnosis towards systemic-pulmonary shunt. Moreover, unfortunately, the blood sample for the SaO_2_ was taken from the middle lobe artery of the right lung. After establishing the diagnosis of idiopathic pulmonary arterial hypertension, we included PAH treatment in accordance with the ESC standards. Control RHC has become a contribution to the diagnosis of multiple system-pulmonary fistulas. Only then did the patient inform us about a right-sided pneumothorax 35 years earlier.

The course of changes in pulmonary hemodynamics can be compared to changes in congenital heart defects leading to the development of the Eisenmenger syndrome but due to the observed clinical improvement, we did not decide to replace iloprost and sildenafil for the recommended endothelin-receptor antagonist [20]. Furthermore, co-occurrence of idiopathic pulmonary arterial hypertension and systemic pulmonary fistulas can’t be clearly excluded.

Finally after many discussions within the heart team, selective embolisation of fistulas was performed. Unfortunately, despite effective embolization, changes in pulmonary circulation were so advanced that removal of the cause of pulmonary hypertension did not improve the patient’s condition. In addition, closure of the fistulas blocked the “*potentially”* beneficial effect of right ventricular decompression in the event of a re-growth of pulmonary artery pressure to super systemic values.

In spite of intensive pharmacological treatment, including the therapy of pulmonary hypertension the patient’s condition deteriorated further. He died three months later due to severe heart failure complicated by pneumonia.

Ji-Feng Li et al. described a similar case of multiple fistulas between systemic and pulmonary arteries of right lung, which were of congenital origin. They resulted in moderate pulmonary hypertension with mean PAP value = 37 mmHg. The right ventricle function was not seriously depressed; TAPSE = 18,5 mm. Embolisation of the largest fistula resulted in a gradual drop of mPAP in six-month follow-up despite the lack of PDE5-I, ARB or prostanoids in concomitant therapy [12].

## Take home message

Non congenital SA-PAFs are extremely rare, but still the diagnosis should be considered when a shunt is suspected during the diagnostic work up of pulmonary arterial hypertension, especially in patients with risk factors.

## Abbreviations

ABPd: Diastolic arterial blood pressure
ABPs: Systolic arterial blood pressure
Angio-CT: Computed tomography angiography
CO: Cardiac output
HIV: Human immunodeficiency virus
HRCT: High-resolution computed tomography
INR: International normalized ratio
mABP: Mean arterial blood pressure
mg: Milligram
mPAP: Mean pulmonary artery pressure
NO: Nitric oxide
NT-proBNP: N-terminal pro-brain natriuretic peptide
O_2_: Oxygen
PAPd: Diastolic pulmonary artery pressure
PAPs: Systolic pulmonary artery pressure
PH: Pulmonary hypertension
ppm: Parts per million
PvO_2_: Venous blood oxygen saturation
PVRI: Pulmonary vascular resistance index
PWP: Pulmonary artery wedge pressure
RAP: Right atrium pressure
RHC: Right heart catheterization
RVSP: Right ventricle systolic pressure
SaO_2_: Systemic blood oxygenation
SA-PAFs: Systemic artery to pulmonary artery fistulas
SVRI: Systemic vascular resistance index
TAPSE: Tricuspid annular plane systolic excursion
TEE: Transesophageal echocardiography
TTE: Transthoracic echocardiography
WHO: World health organization
